# Analysis of Diabetic Foot Deformation and Plantar Pressure Distribution of Women at Different Walking Speeds

**DOI:** 10.3390/ijerph20043688

**Published:** 2023-02-19

**Authors:** Li-Ying Zhang, Qi-Long Liu, Kit-Lun Yick, Joanne Yip, Sun-Pui Ng

**Affiliations:** 1 School of Fashion and Textiles, The Hong Kong Polytechnic University, Hong Kong, China; 2 Laboratory for Artificial Intelligence in Design, Hong Kong Science Park, Hong Kong, China; 3 School of Professional Education and Executive Development, The Hong Kong Polytechnic University, Hong Kong, China

**Keywords:** foot deformation, walking speed, 4D foot scanning, diabetes, plantar pressure

## Abstract

**Highlights:**

- Foot measurements show an insignificant increase at a more rapid walking speed.
- Faster walking speed results in higher mean peak plantar pressure in the forefoot and heel areas, along with a lower pressure time integral in all foot regions.
- Suitable offloading devices are recommended for people with diabetes during exercise at higher walking speeds.
- An insole with a different structure and material for each specific area contributes to plantar pressure offloading.

**Abstract:**

Official guidelines state that suitable physical activity is recommended for patients with diabetes mellitus. However, since walking at a rapid pace could be associated with increased plantar pressure and potential foot pain, the footwear condition is particularly important for optimal foot protection in order to reduce the risk of tissue injury and ulceration of diabetic patients. This study aims to analyze foot deformation and plantar pressure distribution at three different walking speeds (slow, normal, and fast walking) in dynamic situations. The dynamic foot shape of 19 female diabetic patients at three walking speeds is obtained by using a novel 4D foot scanning system. Their plantar pressure distributions at the three walking speeds are also measured by using the Pedar in-shoe system. The pressure changes in the toes, metatarsal heads, medial and lateral midfoot, and heel areas are systematically investigated. Although a faster walking speed shows slightly larger foot measurements than the two other walking speeds, the difference is insignificant. The foot measurement changes at the forefoot and heel areas, such as the toe angles and heel width, are found to increase more readily than the measurements at the midfoot. The mean peak plantar pressure shows a significant increase at a faster walking speed with the exception of the midfoot, especially at the forefoot and heel areas. However, the pressure time integral decreases for all of the foot regions with an increase in walking speed. Suitable offloading devices are essential for diabetic patients, particularly during brisk walking. Design features such as medial arch support, wide toe box, and suitable insole material for specific area of the foot (such as polyurethane for forefoot area and ethylene-vinyl acetate for heel area) are essential for diabetic insole/footwear to provide optimal fit and offloading. The findings contribute to enhancing the understanding of foot shape deformation and plantar pressure changes during dynamic situations, thus facilitating the design of footwear/insoles with optimal fit, wear comfort, and foot protection for diabetic patients.

## 1. Introduction

Diabetes mellitus (DM) is a major health problem globally. The prevalence of diabetes is high, and the number of diabetic patients is still on the rise [1,2]. Diabetic foot ulcers (DFUs) are one of the most common and severe complications of diabetes, which may lead to lower extremity amputation without timely treatment [2,3], and have an immense mental and economic burden on patients [4,5]. The medical treatment of DFUs is expensive and there is still a high risk of recurrence after a DFU has healed [6]. Therefore, early prevention is better than treatment by using various strategies, such as public education [7,8], blood sugar monitoring [8], using offloading devices (footwear/insole) [9], regular foot assessment [10], and physical activity [11], etc. 

Physical activity is inversely associated with the risk of Type 2 diabetes [12,13], and poor physical fitness is regarded as a risk factor for the new-onset of diabetes [14]. Amongst the various types of physical activity, walking is preferred by many people including diabetic patients as their daily activity because it is relatively safe with few side effects [15]. Walking has also been proven effective for weight loss and glucose control for people with diabetes [16,17]. Lakhdar et al. [15] concluded that brisk walking has beneficial effects on anthropometric and biochemical parameters, physical performance, and glycemic control for diabetic patients. However, a fast walking speed can increase the plantar pressure at the forefoot and rearfoot areas [18,19], thereby increasing the risk of DFUs. The pronation of the foot or its side-to-side movement causes the foot to roll a bit inward with each step, with the big and second toes working to push off the foot while the other toes stabilize the foot. The heel strikes the ground, and the arch of the foot flattens and cushions the shock. Then, the foot rolls outward with toe-off, followed by rising and stiffening of the arch as the foot rolls outward and upward. However, a more rapid walking speed may force the foot to a pronated position that increases the plantar pressure at the forefoot [20,21]. Therefore, one of the objectives of this study is to analyze the plantar pressure changes of diabetics at different walking speeds, in order to design appropriate offloading insoles/footwear to protect their feet from high levels of pressure during daily physical exercise.

Insole geometry is an important factor in the redistribution of plantar pressure [22]. The shoe/insole must align with the shape of the foot with different stances for an appropriate fit [23]; otherwise, an abnormal in-shoe pressure would result. Therefore, accurate and reliable foot anthropometric measurements can provide valuable information for shoe last/insole geometry designs. Previous studies have concluded that the geometry of the foot changes with different loads in both standing and walking conditions [24,25,26]. Xiong et al. [24] and Zhang et al. [27] stated that the foot increases both in length and width, and reduces in height with increases in weight bearing. The shape of the foot also becomes wider at the forefoot and heel during roll-over compared to a static stance [23]. On the basis of these findings, a wider toe box and insole with arch support are recommended to provide a better fit and prevent foot injury caused by a mismatch of the different foot dimensions. The heel cup of a foot orthosis also can add to the thickness of the heel fat pad, which can further reduce the peak plantar pressure by increasing the contact area during standing and walking [28,29]. In addition, the heel cup can also limit the deformation of plantar soft tissues [29]. However, these previous findings have mainly focused on foot deformation during static conditions or one walking speed only. To date, studies on foot deformation at different walking speeds have been few and far in between.

Footwear research has investigated the biomechanical changes of the lower limbs at different walking speeds [30,31,32] and found that an increased speed of gait results in greater ground reaction forces (GRFs). The shape of the foot tends to easily deform when walking at different speeds with changes in loading. The shape of the foot therefore provides important information for shoe last modelling which is closely associated with footwear design and fit [33]. Footwear is the protective measure of the foot during motion, and studies such as [34] have concluded that foot shape must be taken into consideration in footwear design so as to prevent negative effects on foot morphology. However, the shoe last construction currently is mainly based on individual practical knowledge which relies on trial-and-error [23]. Therefore, it is essential to investigate the deformation of the foot geometry in dynamic situations at different walking speeds to gain more knowledge towards the design of shoe lasts. Another objective of this study is to investigate the effect of the walking speed on changes in the geometry of the diabetic foot during walking so that offloading devices with optimal fit can be precisely designed and prescribed.

The objectives of this study are as follows:

(1) to analyze the effect of walking speed on plantar pressure distribution in dynamic situations;
(2) to investigate the effect of walking speed on the deformation of the foot geometry.

## 2. Materials and Methods

### 2.1. Participants

A total of 19 female subjects who ranged from 57 to 75 years old (mean: 66, SD: 5) participated in the study. They self-reported Type 1 or 2 diabetes mellitus (DM) in the early stages (based on diagnosis of a clinical physician). The inclusion criteria [35,36] were those with no history of ulcers or neurological disorders (except neuropathy) and who had the ability to walk a length of 20 m repeatedly without any walking aid. Those who showed the presence of active foot ulcers and severe foot deformities that would affect gait were excluded [37]. All of the participants provided written informed consent after they were given an introduction of the experimental procedures and requirements. The experiment protocols were approved by the Human Subjects Ethics Sub-Committee of the University (Reference Number: HSEARS20200128001).

Before conducting the experiment, a preliminary short survey was conducted on a face-to-face basis with participants to collect first-hand information about the physical activity habits to maintain health in their daily life, as well as features and problems with regard to wearing footwear during their physical activities. The responses showed that most of them preferred walking in their daily activity, and footwear discomfort was found in different foot areas.

### 2.2. Experiment Protocols

All of the subjects were required to walk on the walkway (4.5 m in length) of a foot scanning system (3dMD LLC, Atlanta, GA, USA) at 3 different walking speeds. The normal walking speed was defined as the natural pace of each subject, and the fast and slow speeds of walking were 20% faster and 20% slower than the natural walking speed, respectively [38]. Two timing gates (Brower Timing System, Draper, UT, USA) were placed at each end of the walkway to determine the duration of each trial. The experiment was divided into 3 sections:

(1) First, the subjects walked on the walkway 10 times at their natural pace to determine their normal speed and calculate the slow and fast walking speeds. Scanning and plantar pressure measurement trials that exceeded 5% of the predetermined speeds were rejected to minimize the effect of speed on foot deformation and plantar pressure distribution.
(2) Then, both the left and right feet were scanned 3 times at the three walking speeds defined in the first step.
(3) The plantar pressure of the subjects in their bare feet at the 3 defined walking speeds was recorded 3 times, and the subjects wore standard cotton socks to secure Pedar sensors onto the plantar of their feet.

**Figure 1:**
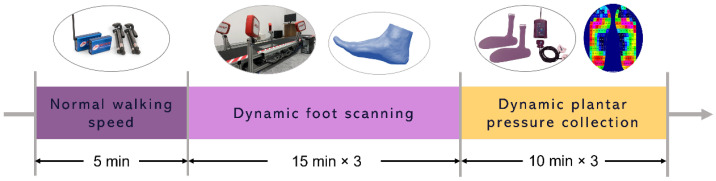
Experiment flow.

The subjects were given a break of 1 min after 3 scans. The 3 walking speeds were randomized to minimize the order effects. The experimental flow is shown in Figure 1.

### 2.3. Foot Image Analysis

**Figure 2:**
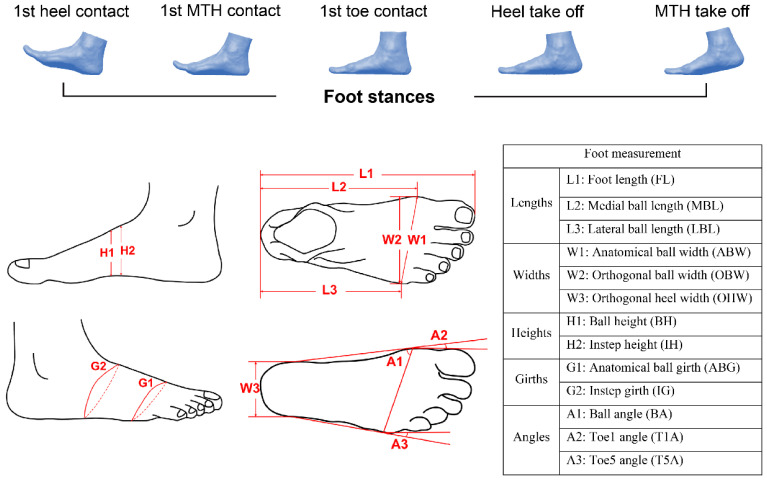
Five frames of foot stances and foot measurements.

The stance phase was divided into 5 frames: at first heel contact, first metatarsal head (MTH) contact, first toe contact, heel take off, and MTH take off, respectively, [25,26] based on the defined functional time frames of the foot during roll-over (see Figure 2). Thirteen relevant foot anthropometric measurements of each frame including the lengths, widths, heights, girths, and angles were extracted for an in-depth analysis [25]. 

### 2.4. Plantar Pressure Analysis

**Figure 3:**
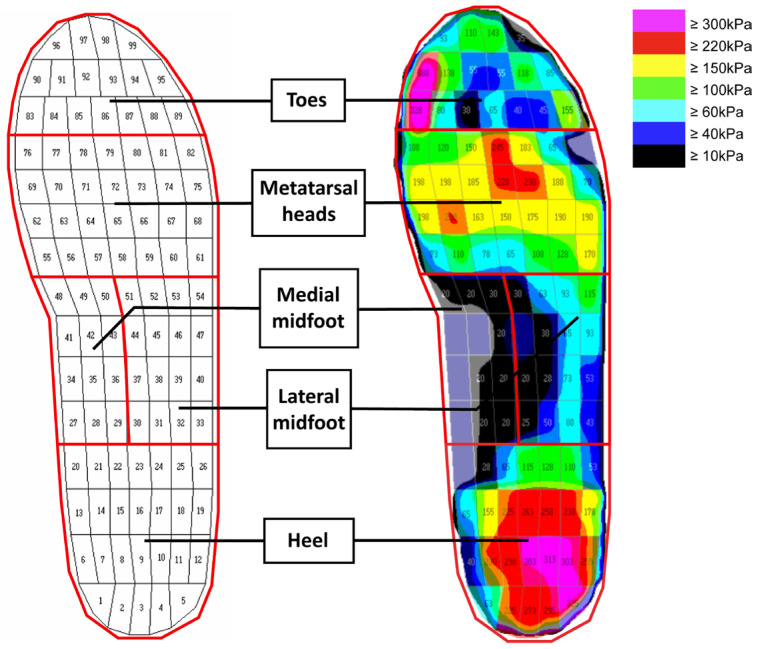
Five plantar areas of the foot.

The plantar of the foot was divided into 5 areas for the plantar pressure analysis: toes, metatarsal heads, medial midfoot, lateral midfoot, and heel (see Figure 3). The mean peak plantar pressure (MPP) and pressure time integral (PTI: accumulation of foot pressure during plantar contact time) of each analyzed area of the foot during the 3 different walking speeds were used to describe the foot biomechanics.

### 2.5. Data Analysis

All of the foot measurements and pressure parameters were analyzed by using SPSS Statistics 21 software (IBM Corp., Armonk, NY, USA). A Shapiro–Wilk test was used to determine the normality of the 13 foot measurements and plantar pressure obtained for each measured part of the foot at the 3 different walking speeds. The results showed that all of the measurements and parameters were normally distributed (*p* > 0.05). A one-way repeated measures analysis of variance (ANOVA) was used to compare the mean value of each foot measurement and plantar pressure measured at the 3 walking speeds, and to determine whether there were significant (*p* < 0.05) differences.

## 3. Results and Discussion

### 3.1. Participant Information

The description statistics of the participants in this study are listed in Table 1, including their age, body mass index (BMI), foot size, and years since diagnosis of DM.

### 3.2. Effect of Walking Speed on Foot Deformation

The maximum value of each foot measurement from the five selected frames was extracted for analysis. The one-way repeated measures ANOVA showed that there is no significant difference for each foot measurement at the three walking speeds. Table 2 shows that each foot measurement slightly increases with a more rapid walking speed. The increase (in percentage) of the toe angles and orthogonal heel width is larger than that of the other foot measurements.

**Figure 4:**
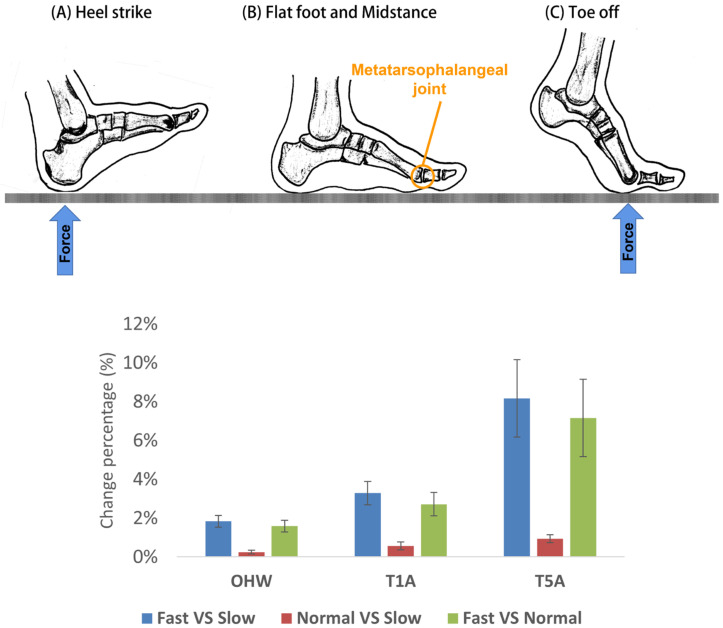
Key phases of the roll-over process: (**A**) heel strike, (**B**) flat foot and midstance, (**C**) toe-off.

The walking speed shows a positive effect on all of the foot measurements. All of the foot measurements increase at a more rapid walking speed due to the larger GRFs associated with a fast gait speed [39,40]. The medial arch plays an important role in shock absorption and transfer of weight bearing during walking or running [41]. Deformation occurs in the medial arch during walking, including extension, pull-up, and collapse of the medial arch. The toes control the height of the arch during walking, so more toe flexion means a higher pull-up of the foot arch and extension of the longitudinal arches. Caravaggi et al. [40] concluded that the metatarsophalangeal (MTP) joints show more dorsiflexion when walking at a fast pace, and the arch height and foot lengths thereby increase with speeding up the gait. During midstance in a gait cycle, the forefoot pronates with the medial arch stretched and flattened to store mechanical energy for propulsion. The ball angle (BA) thereby increases with speeding up the gait. It is found in this study that the measurements of the forefoot and heel areas show relatively larger deformation with increased walking speeds as opposed to the midfoot. During roll-over, only the forefoot and heel have contact with the ground at the beginning and end of the stance phase, of which the GRFs peak; see Figure 4 [23]. The foot measurements increase with larger loads imposed on the foot; therefore, greater deformation of the foot shape occurs with speeding up of gait [27], especially at the forefoot and heel.

Regarding these foot deformations, shoes for daily wear may not best fit the foot shape during exercise with a rapid gait. Shoes with an arch-conforming shape are recommended to prevent the increase in stretch of the arch for pressure offloading during walking at a rapid pace [42]. In addition, a wider toe box should be used for diabetic footwear to accommodate the broadened forefoot with increases in walking speed. A heel cup in running shoes is also recommended to minimize the deformation of the soft tissues in the heel of the plantar.

### 3.3. Effect of Walking Speed on Plantar Pressure Distribution

The MPP and PTI are commonly used measures to assess the foot biomechanics with different movements and related to foot pain and the development of foot ulcers in diabetic patients [32]. The plantar of the foot in this study is divided into five specific areas: toes, metatarsal heads, medial midfoot, lateral midfoot, and heel. After implementing a one-way repeated measures ANOVA, the results showed that the MPP increases significantly at a faster walking speed except at the midfoot; see Table 3. However, the PTI decreases significantly with the exception of the medial midfoot and heel areas. 

**Figure 5:**
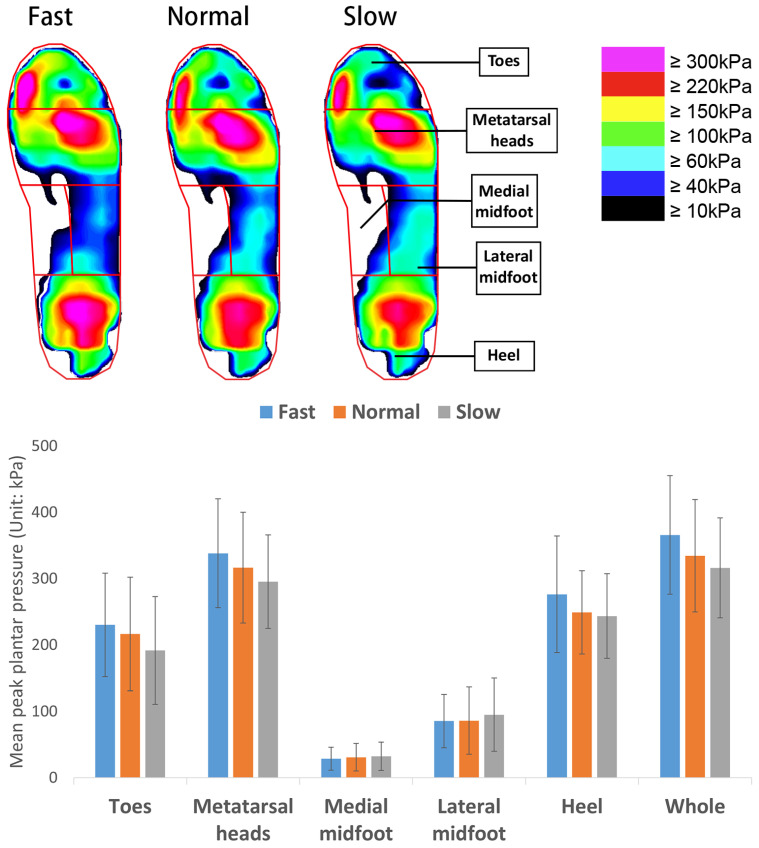
Mean peak plantar pressure at 3 walking speeds.

**Figure 6:**
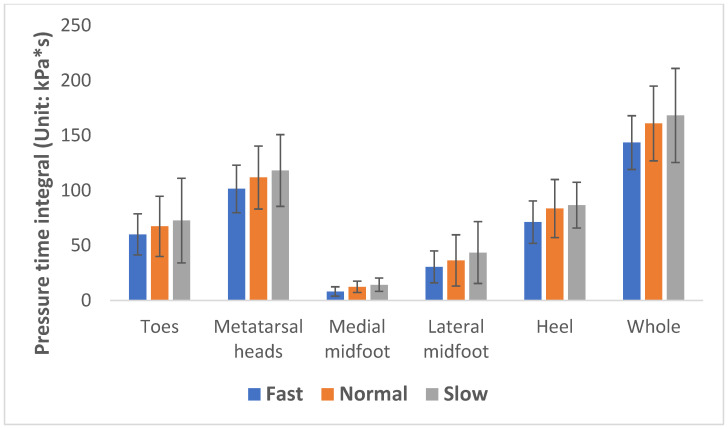
Pressure time integral at 3 walking speeds.

As shown in Figure 5 and Figure 6, regardless of whether there is a significant difference, the walking speed has a positive effect on the MPP with the exception of the midfoot, while it shows a negative effect on the PTI. The MPP increases along with a decreased PTI at a more rapid walking speed. This can be attributed to the fact that a faster walking speed is associated with larger GRFs [31,40,43] with a similar amount of foot plantar contact, so higher pressure is found in most of the foot regions. Moreover, a faster gait speed also has a shorter total contact time, thus resulting in a lower PTI [40,44,45].

However, higher peak plantar pressure at a more rapid walking speed and higher accumulation of plantar pressure at a slower pace are found in this study, which might lead to skin breakdown and foot pain. Previous studies have concluded that walking at a rate of 4 km/h and wearing an ethylene-vinyl acetate (EVA) insole can reduce plantar pressure at the forefoot [46]. A heel pad with an auxetic structure has been proven to reduce the pressure in the heel area during walking [47]. Chen et al. [48] also recommended dividing the plantar of the foot into support and soft areas, so that honeycomb and auxetic structures for support and pressure offloading could be applied, respectively. Therefore, suitable offloading devices combined with appropriate speeds of walking are recommended for DM patients to maintain fitness and minimize their risk of DFUs. 

While walking is the most common physical activity because it can be done anywhere and requires no equipment, there are still reservations about promoting physical exercise for DM patients, and sometimes the advice given is “rest to avoid injury”. However, studies show evidence that exercise does not increase the incidence of ulceration and re-ulceration of DM patients [49,50]. The foot serves as the basis for locomotion, and effective push off from the ground requires the MTP joints to undergo substantial flexion and extension [51]. A larger MTP dorsiflexion angle is found with faster walking speeds [52]. However, insufficient MTP joint dorsiflexion may shorten the step length, and this gait pattern may increase the risk of falls and injury [53]. Therefore, flexibility exercises that focus on the foot such as manually mobilizing the forefoot into dorsiflexion and brisk walking are recommended for DM patients to maintain or improve their range of motion in the ankle and foot. Appropriate walking speeds while using a suitable insole will help to increase the range of motion of the foot and reduce joint stiffness and peak foot pressure. It is therefore essential to take extra good care of diabetic feet during and after walking rather than eliminating exercise. Considering the clinical implications of the results of this study, plantar pressure changes with different walking speeds would be useful to recommend activities for patients with specific foot problems. For example, patients with forefoot pain should slow down, since a fast walking speed needs more range of motion of the first metatarsophalangeal joint, which increases the shear force contributing to skin breakdown [18,54].

Having said that, there are some limitations of this study. First, only 19 female subjects are involved, which is a fairly small sample size and limits the generalizability of the results. Future studies can involve a larger number of subjects. Despite the limitations, this study gives insight into the changes in the foot geometry and pressure at different walking speeds, thereby acting as a reference for studies that aim to optimize the wear comfort of diabetic footwear and insoles during daily physical exercise.

## 4. Conclusions

In summary, each foot measurement in this study increases with acceleration during locomotion, but the effect of the walking speed is insignificant. The shapes of the forefoot and heel show a larger percentage of size increase as opposed to the midfoot. Meanwhile, a faster walking speed significantly increases the mean peak pressure at the forefoot and heel areas. However, the PTI of each area of the foot decreases with the acceleration in gait, which suggests that a slower pace of walking cannot reduce the pressure accumulation with ground contact. Therefore, suitable offloading devices are recommended for diabetics during walking to prevent foot injury due to high pressure. Design features such as a wide toe box, medial arch support, and inclusion of heel cups are recommended to optimize the fit of the footwear for walking. Furthermore, custom-fabricated insoles with conforming 3D arch support and/or cushioning heel pads made of a plurality of materials such as ethylene vinyl acetate, polyethylene, etc., can help to redistribute the plantar pressure, hence reducing the risk of foot ulcers and preserving the mobility of diabetic patients.

## Figures and Tables

**Table 1:** Description statistics of participants (*n* = 19).

Variable	Mean	Standard Deviation	Maximum	Minimum
Female (*n* = 19)				
Age (years old)	66	5	75	57
BMI (kg/m^2^)	22.3	3.2	30.8	18.2
Foot size (EUR)	38	1	41	37
Years since diagnosis (DM)	13	1	31	10

**Table 2:** ANOVA analysis of foot measurements at slow, normal, and fast walking speeds.

Foot Measurement (mm)	Mean (Standard Deviation)			Fast vs. Slow (%)	Normal vs. Slow (%)	Fast vs. Normal (%)
	Slow	Normal	Fast			
Foot length (FL)	237.0 (9.3)	237.2 (8.5)	237.5 (9.0)	0.2%	0.1%	0.2%
Medial ball length (MBL)	177.1 (4.3)	177.1 (4.8)	178.5 (5.5)	0.8%	0.0%	0.8%
Lateral ball length (LBL)	152.4 (4.8)	152.4 (3.7)	153.8 (4.2)	0.9%	0.0%	0.9%
Anatomical ball width (ABW)	98.8 (4.9)	98.9 (5.3)	99.4 (4.8)	0.6%	0.1%	0.5%
Orthogonal ball width (OBW)	92.8 (4.3)	92.8 (4.8)	93.5 (4.5)	0.7%	0.0%	0.7%
Orthogonal heel width (OHW)	52.0 (4.4)	52.1 (4.2)	53.0 (5.8)	1.8%	0.2%	1.6%
Instep height (IH)	61.8 (3.3)	61.9 (3.7)	61.9 (2.8)	0.2%	0.1%	0.1%
Ball height (BH)	47.8 (3.7)	47.9 (3.8)	47.9 (2.3)	0.3%	0.3%	0.0%
Ball angle (BA)	78.4 (9.1)	79.0 (9.7)	79.1 (9.6)	0.8%	0.7%	0.1%
Toe1 angle (T1A)	17.2 (5.4)	17.3 (5.9)	17.7 (5.3)	3.3%	0.6%	2.7%
Toe5 angle (T5A)	13.6 (3.1)	13.7 (3.5)	14.7 (2.3)	8.2%	0.9%	7.2%
Anatomical ball girth (ABG)	225.6 (11.5)	226.0 (13.1)	226.2 (13.3)	0.3%	0.2%	0.1%
Instep girth (IG)	238.3 (11.9)	238.4 (13.0)	239.1 (14.7)	0.3%	0.1%	0.3%

**Table 3:** ANOVA of pressure measurements at slow, normal, and fast walking speeds.

Area of Pressure Measurement	Mean (Standard Deviation)			Fast vs. Slow (%)	Normal vs. Slow (%)	Fast vs. Normal (%)
	Slow	Normal	Fast			
**Mean peak pressure (kPa)**						
Toes	191.8 (80.9)	216.6 (85.6)	230.2 (77.9)	**20.0%**	**13.0%**	6.3%
Metatarsal heads	295.4 (70.4)	316.4 (83.6)	338.1 (82.1)	**14.5%**	**7.1%**	**6.9%**
Medial midfoot	32.5 (21.4)	30.9 (20.6)	28.7 (17.3)	**−11.7%**	**−4.7%**	−7.3%
Lateral midfoot	94.9 (55.1)	86.0 (50.7)	85.5 (40.4)	−9.9%	−9.4%	−0.6%
Heel	243.5 (63.9)	249.2 (62.7)	276.4 (88.0)	**13.5%**	2.4%	**10.9%**
Whole	316.1 (75.3)	334.4 (84.7)	365.9 (89.4)	**15.8%**	5.8%	**9.4%**
Pressure time integral (kPa*s)						
Toes	72.7 (38.4)	67.4 (27.3)	60.2 (18.6)	**−17.2%**	**−7.2%**	−10.8%
Metatarsal heads	118.3 (32.6)	111.9 (28.7)	101.5 (21.6)	**−14.2%**	−5.4%	**−9.3%**
Medial midfoot	14.4 (6.1)	12.5 (5.1)	8.3 (4.2)	−42.6%	−12.9%	−34.1%
Lateral midfoot	43.6 (28.1)	36.5 (23.3)	30.6 (14.5)	**−29.9%**	−16.4%	**−16.1%**
Heel	86.7 (20.8)	83.7 (26.4)	71.2 (19.3)	−17.8%	−3.5%	−14.9%
Whole	168.3 (42.8)	161.1 (34.0)	143.6 (24.4)	**−14.7%**	−4.3%	**−10.8%**

* Significant differences at the 0.05 level are bolded.

## Data Availability

The datasets used and/or analyzed during the current study are available from the corresponding author on reasonable request.
